# Ontogeny of the digestive system of the *Octopus bimaculatus* paralarvae (Verril, 1883)

**DOI:** 10.1186/2193-1801-3-22

**Published:** 2014-01-10

**Authors:** Diana Judith López-Peraza, Mónica Hernández-Rodríguez, Benjamín Barón-Sevilla

**Affiliations:** Centro de Investigación Científica y de Educación Superior de Ensenada (CICESE), Baja California, Mexico

**Keywords:** Ontogeny, Digestive system, Paralarvae

## Abstract

The high mortalities registered in the larval stage during octopus culturing are mainly due to nutritional deficiencies of the food provided. To understand the cause of this problem, we studied the ontogenetic development of the digestive system of *Octopus bimaculatus* paralarvae. An egg batch was obtained from a gravid female collected in the Bay of Los Angeles, Baja California, Mexico, and it was incubated in the laboratory during the summer of 2011. We observed that the formation of the digestive system began at 33 days post-laying (DPL). The newly hatched paralarvae had already formed the organs involved in food ingestion and digestion, although it was not possible to know accurately their degree of maturity. The present research constitutes the first description at the histological level of the ontogenic development of the digestive system of the *O. bimaculatus* paralarvae. This serves as a basis for future studies of the digestive physiology of this species.

## Introduction

Embryonic development of the octopus follows two main patterns, one of them, culminating in the hatching of a body of benthic habits, with characteristics very similar to those of an adult (e.g. *Octopus maya*) and they do not go through a process of metamorphoses after hatching as in the case of many marine fish. In other species, such as *Octopus vulgaris* and *Octopus bimaculatus*, embryonic development culminates with the hatching of a planktonic organism, known as “paralarva” (Young and Harman [1988]).

Culturing *O. maya* has developed relatively quickly on the coast of Campeche, Mexico (Duhne [2010]) and although knowledge of their biology is still limited, it is likely that the success is due to the juveniles hatch with a greater degree of maturity of the digestive system and readily accept the first food, which generally includes adult brine shrimp and live palemonids and fragments of frozen crustaceans (Rosas et al. [2006]). In contrast, the development of the culture of *O. vulgaris* has been slowed by the low or no survival of their paralarvae, since the newly hatched planktonic paralarvae die during the first days after hatching.

For this reason, in Spain and Peru, the commercial cultivation of octopus has been limited to the capture of wild juveniles for fattening in cages or special devices (Iglesias et al. [1997]; Moxica et al. [2002]). This limitation in the cultivation of this species of economic importance to Spain, has prompted an intense research effort to understand the biology of the early stages of development, their nutritional needs, a live diet adequate in size, quantity and nutrient composition and finally, the standardization of culturing techniques (Moxica et al. [2002]; Iglesias et al. [2004]; Iglesias and Sánchez [2007]).

During larval stages of development, ontogeny involves major changes in the structure and function of tissues, organs and systems (Zambonino-Infante and Cahu [2001]). Thus, comparative studies of the digestive system in different stages of development can serve as a framework for studies of digestive physiology of organisms in which the development of culture techniques have been limited by the problems represented by the initial feeding which results in very high mortality. This information is useful to select the diet that meets the nutritional requirements of the paralarva that as in fish larvae, their eating habits must be correlated with the structure of the digestive system (Luizi et al. [1999]; Roo et al. [1999]; Izquierdo et al. [2000]).

Morphological and histological analysis of organs that make up the digestive system of marine fish larvae, have been widely used as a tool for studying its functionality. Where changes in cell structure after the larva begins exogenous food intake may be related to the degree of maturity of these organs during different stages of development (Osman and Caceci [1991]; Boulhic and Gabaudan [1992]; Segner et al. [1994]; Bisbal and Bengtson [1995]; Sarasquete et al. [1995]; Ribeiro et al. [1999]. Elbal et al. [2004]; Gisbert et al. [2004]).

Most existing work on the biology of embryonic development of cephalopod paralarvae have focused on various species of squid, particularly the genus *Loligo* (Arnold [1965]; Marthy [1975]; Asokan and Kakati [1991]; Barón [2002]; Cardoso et al. [2005]), species of the genus *Illex* (O’Dor et al. [1982]; Boucaud-Camou and Roper [1998]; Villanueva et al. [2011] and *Sepioteuthis* (Alagarswami [1966]). Boletzky ([2003]) conducted a comprehensive study of the biology of the early stages of cephalopods, in which species of squid, cuttlefish and octopus are included. Studies on the biology of embryonic development of paralarvae, regarding octopuses, have been carried out over a very few species and these belong to the genus *Octopus* (Marthy [1975]; Boletzky [2003]; Ignatius and Srinivasan [2006]; Ávila-Poveda et al. [2009]). However, none of these studies have described the development of the organs that make up the digestive system of the paralarvae.

Knowledge about developmental biology and digestive physiology of their paralarvae is insufficient and even zero in some species, such as the case of the spotted octopus *O. bimaculatus*. Because this species with economic value and potential of culture is very important for Mexico, and that is why is of fundamental importance to understand the development of paralarva, especially the organization and function of the digestive system. That is why the purpose of this study was to describe histologically the organs that make up the digestive system in the different phases of ontogenetic development of the paralarvae of *O. bimaculatus* and provide a basis for future research on the digestive physiology of this species.

## Material and methods

Fourteen females of *O. bimaculatus* were collected in the Bay of Los Angeles, BC, Mexico, located between 29° 02’ and 28° 57’ N and 113° 32’ and 113° 26’ W (INEGI [2013]). The organisms were transported to the Aquaculture Department of the Centro de Investigación Científica y de Educación Superior de Ensenada, BC (CICESE) in a plastic container with a capacity of 1 m^3^, filled to 50% with the sea water of the Bay. During transport, the dissolved oxygen concentration remained up to 8 mg·L^-1^, with constant pure oxygen injection. In order to avoid aggression between octopuses during the transport, each one was placed in individual refuges made with ABS pipe 15.2 cm in diameter and 20 cm in length, and each tube was placed in a bag made with sardine mesh moored at the ends.

Once in the laboratory, the females were individually placed in 500 L plastic tanks with continuous sea water replacement at a 200% daily rate, regular aeration and without temperature control. A clay pot was placed as a refuge. The physicochemical parameters of the water were measured daily. Only one of the females lay eggs and during the incubation, the female cared for them, which assured good development until paralarvae hatching. During this research, the ethical recommendations for handling laboratory octopuses by Moltschaniwskyj et al. ([2007]) were followed. In order to study the ontogeny of the organs that integrate the digestive system, daily samples (n = 15) were taken of the eggs, from the beginning of laying (0 days post-laying, DPL) until the day of hatching (0 days post-hatching, DPH). The samples were fixed in Davidson’s solution in a relation sample-fixation of 1:5.Later, the samples were dehydrated in a Histokinette processor Leica model TP1040. Then, they were embedded in paraffin, by means of a paraffin dispenser Leica model EG1160. Once the paraffin blocks were formed, longitudinal cuts in sections of 5 μm of complete eggs and newly hatched paralarvae were made with a microtome American Optical Spencer-820-320. In order to contrast tissues, the technique of Arteta trichromic stain (Valderrama et al. [2004]) was used. The samples were analyzed and captured by means of a microscope Olympus model CKX41, equipped with an Olympus camera C-5060 model. Finally, the organs and tissues that integrate the digestive system of paralarvae were identified, described histologically and their relation to each other was established, taking as reference the digestive system from an adult of *O. bimaculatus*.

## Results

**Figure 1:**
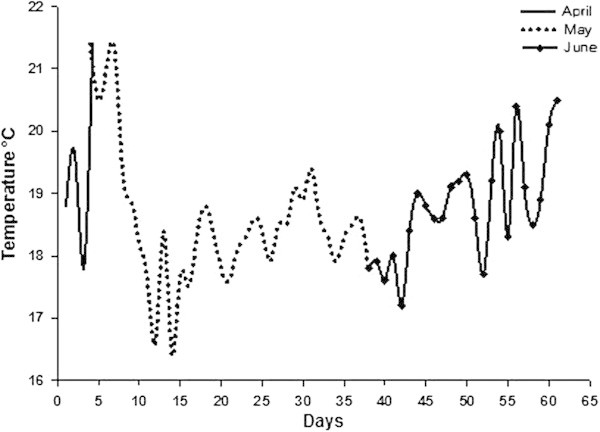
**Eggs incubation temperatures of** ***Octopus bimaculatus*** **from April to June 2011.**

The incubation of the eggs lasted 61 days. During that time, the variation of the water temperature was from 16.5 to 21.5°C from April to June of 2011 (Figure 1).

### Ontogeny of the digestive system during the embryonic development

**Figure 2:**
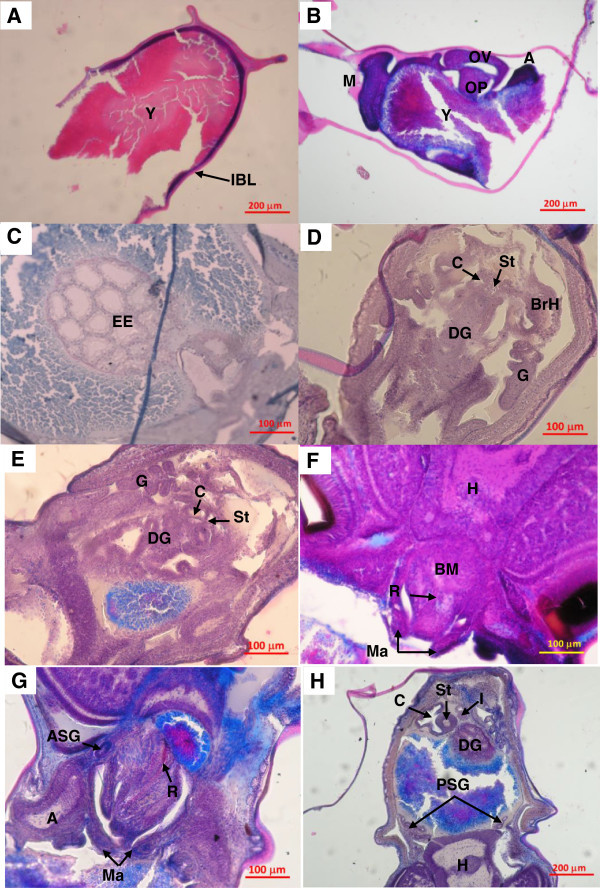
**Longitudinal cuts of** ***O. bimaculatus*** **eggs. A**, embryo of 20 DPL, with extended blastodermeres towards the anterior part of the egg on the yolk surface. **B**, embryo of 30 DPL, with early mantle, eyes and arms differentiation. **C**, ectodermal stomodeum in the anterior part of an embryo at 33 DPL. **D**, outlines of the internal organs of an embryo at 37 DPL. **E**, embryo at 39 DPL, section of the internal cavity of the mantle. **F**, embryo at 44 DPL, buccal mass. **G** and **H**, embryo of 55 DAL anterior and posterior sections respectively. Arteta trichromic stain. A, arm; ASG, anterior salivary gland; BrH, branchial heart; BM, buccal mass; C, caecum; DG, digestive gland; EE, ectodermal stomodeum; G, gills; H, head; I, intestine; IBL, invagination of the blastoderm; M, mantle; Ma, mandible; OP, ocular peduncle; OV, optical vesicle; PSG, posterior salivary gland; R, radula; St, stomach; Y, yolk.

The embryo development of *O. bimaculatus* began with the proliferation of cells (blastomeres) on the external surface of the yolk on the animal pole of the egg. Later, this layer of cells extended surrounding the outer surface of yolk, towards the anterior of the egg, where the peduncle attaches to the eggs cluster. At 20 DPL, the blastoderm invagination that gives rise to the eyes began to form (Figure 2A). The thickness of the blastoderm, in this zone, diminished from 5 to 2 cells. In this stage the blastoderm covered approximately 50% of the egg surface; nevertheless, the embryo did not yet experience the first inversion. In subsequent stages (27 DPL) the mantle, eyes and arms outlines were formed. Later (30 DPL), a greater degree of development of these structures was observed, particularly of the eyes, when it was possible to observe the protrusion of the eyestalk and the optical vesicle, the last one constituted by an undifferentiated stratified epithelium (Figure 2B). Also at 30 DPL, more than 50% of the embryos had experienced the first inversion towards the anterior part of the egg.

At 33 DPL, the invagination of the blastoderm, known as ectodermal stomodeum, was observed in the anterior section of the embryo (Figure 2C), which indicated the beginning of the formation of the structures that constitute the buccal mass and the anterior esophagus. From 37 DPL, the outlines of the stomach, the caecum and the digestive gland were observed. The stomach and the caecum were formed by stratified epithelium without cellular differentiation. The digestive gland did not have the secretory tubules defined or the epithelium that covers them. In this stage, also the primordium of gills and branchial hearts were observed (Figure 2D). In addition, the embryos had three suckers in each arm. The stomach and the caecum in the embryo of 39 DPL were observably larger, although the inner stomach epithelium was not yet totally differentiated. The digestive gland was larger and the secretory tubule and epithelium that covers it developed. The lumen of some tubules was wide and the spaces between each tubule were clearly visible (Figure 2E). At 44 DPL, a greater degree of development in the structures that comprise the buccal mass were observed, particularly the mandibles. The buccal mass was perfectly delimited and surrounded by muscular fibers, and in the internal cavity the outline of the radula was evident (Figure 2F).

At 55 DPL, more than 50% of the embryos experienced the second inversion and the viteline reserve diminished considerably, compared to the early stages. The structures that formed the buccal mass, i.e., mandibles, radula, and anterior salivary glands, already resembled newly hatched paralarvae (Figure 2G). The posterior salivary glands were located in the anterior portion of the internal cavity of the mantle, just near the head. The stomach increased its size and its lumen was better defined; however, the epithelium of this organ still was undifferentiated in comparison to newly hatched paralarvae. The caecum displayed a simple cylindrical epithelium very folded, with long cilia oriented towards the lumen. The digestive gland had a greater size, and there was greater development of the secretories tubules, which are constituted by a simple epithelium of ciliate cells, and the space between them reduced. Also a section of the intestine was observed, which was constituted by a columnar epithelium (Figure 2H).

### Description of the digestive system of paralarvae newly hatched (0 DPH)

**Figure 3:**
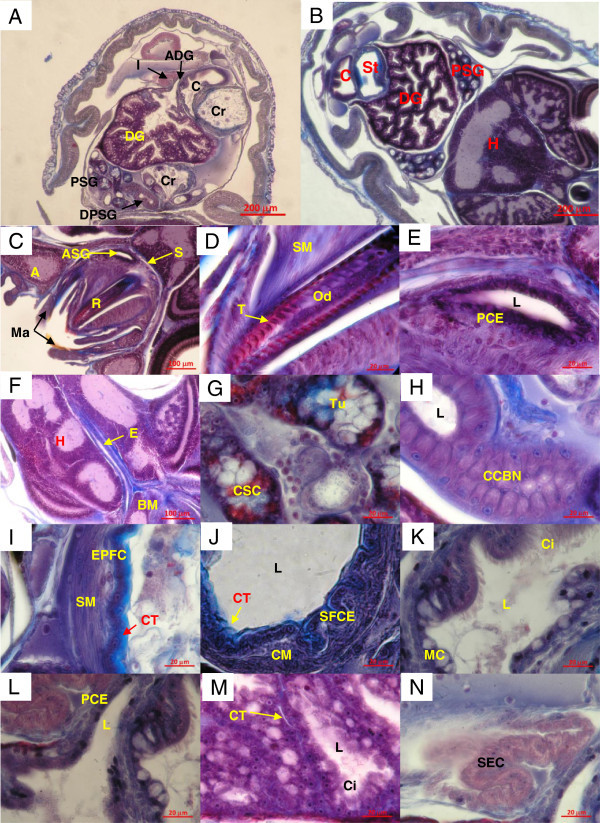
**Longitudinal cuts of the digestive system of** ***O*** **.** ***bimaculatus*** **paralarvae at 0 DPH.** **A** and **B**, location of the organs that integrate the digestive system in the internal cavity of the mantle. **C**, buccal mass. **D**, radula. **E**, detail of the anterior salivary gland. **F**, esophagus embedded in the cartilaginous structure of the paralarvae head. **G**, detail of the secretory tubules of the posterior salivary gland. **H**, augmentation of the duct that communicates to the two sections of the posterior salivary gland with the buccal mass cavity. **I**, detail of the crop structure. **J**, approach of stomach epithelium. **K**, structure of caecum epithelium. **L**, appendix of the digestive gland. **M**, secretory tubules of the digestive gland. **N**, intestinal epithelium. A, arm; ADG, appendix of the digestive gland; ASG, anterior salivary gland; BM, buccal mass; C, caecum; Ci, cilia; CCBN, columnar cells with basal nuclei; CM, circular muscle; Cr, crop; CSC, cubical secretory cells; CT, connective tissue; DG, digestive gland; DPSG, duct of the posterior salivary gland; E, esophagus; EPFC, epithelium of pseudostratified flat cells; H, head; I, intestine; L, lumen; MC, mucous cells; Ma, mandible; Od, odontophore; PCE, pseudostratified columnar epithelium; PSG, posterior salivary gland; R, radula; S, sine; SEC, simple columnar epithelium; SM, smooth muscle; SFCE, simple epithelium of flat cells; St, stomach; T, teeth; Tu, tubule.

Hatching was registered at 61 DPL. Newly hatched paralarvae had the organs involved in the ingestion and digestion of food (Figure 3A and B). The outer region of the buccal mass (Figure 3C) had two mandibles, surrounded by a close sine that facilitated its movement. The radula (Figure 3D), located in the buccal mass cavity, was formed by rows of teeth parallel to the main axis of the radula, and each row had 30 longitudinal quitina teeth positioned parallel. The radula was anchored to the odontophore which was constituted by a columnar epithelia. The anterior salivary glands were located on the distal portion of the buccal mass (Figure 3C). These organs are constituted by pseudostratified epithelium of columnar cells (Figure 3E).

The buccal cavity was directly related to the esophagus, which is a very narrow long tubule. This organ initiated in the buccal mass cavity, crossed the skull (by analogy with the vertebrates) and ended behind the head. Histologically, its external wall was surrounded by extended straight muscular fibers, whereas the inner part, near the lumen, was formed by muscular fibers arranged in circular form (Figure 3F). It is possible that this disposition facilitates the ingested food transport by contraction of the esophageal regions, throughout the whole structure, until arriving at the crop. In the anterior portion of the crop, adjacent to the brain, were the posterior salivary glands. These glands were formed by cubical secretory cells with apparent mucus secretion (Figure 3G). In accordance with the affinity of the cellular types to the dyes used, it was deduced that there were different types of secretions. The secretions of the glands were transported directly to the buccal mass cavity of the organism through a tube that ran parallel to the esophagus (observation in an adult of *O. bimaculatus*). This tube was formed by cubical cells with basal nuclei (Figure 3H).

The crop was a voluminous sac surrounded by straight muscular fibers; internally it was covered by pseudo-stratified epithelium of flat cells (Figure 3I). The internal epithelium had abundant collagen fibers towards the lumen. Immediately and connected to the crop, the stomach was found (Figure 3J); its lumen was covered by a simple epithelium of flat cells very folded and abundant connective tissue, mainly of collagen fibers. The external wall was made up of a thick layer of muscle fibers arranged in circular position. The stomach was also connected with the caecum (Figure 3K) that was of spiral shape; towards the lumen it had a simple cylindrical epithelium with long cilia and mucous cells. The caecum was connected with the digestive gland through two appendixes of the digestive gland also called pancreatic appendix. These appendixes were thin ducts constituted by a pseudo-stratified epithelium of columnar cells (Figure 3L) and by means of which, the digestive gland poured its secretions towards the caecum. The digestive gland was the organ of greatest volume in paralarvae. It was delimited by a thin membrane and its interior was constituted by secretory tubules with a simple cylindrical epithelium of ciliate cells. Each tubule was surrounded by connective tissue of collagen fibers (Figure 3M).

The intestine was an extended duct in direct bond with the caecum and it was folded in the mantle cavity. It ended in the distal opening or rectum located near the distal portion of the ink sack, both adjacent to the siphon. Histologically, it was constituted by a simple columnar epithelium (Figure 3N).

## Discussion

Studies related to the ontogeny and the cultivation of the octopus agree that, during the paralarvae stage, a mortality rate higher than 50% is registered, which mainly is attributed to the transition between the consumption of the yolk in the embryonic phase and the beginning of exogenous feeding, which involves the ability of the paralarvae to find and capture food and its capacity to digest it (Moxica et al. [2002]; Iglesias et al. [2004]; Iglesias and Sánchez [2007]). The present research is the first study on the ontogeny of the organs and structures that comprise the digestive system of *O. bimaculatus* paralarvae. Like many diverse marine oviparous species, the embryonic development of paralarvae of the cephalopods is highly dependent on the water temperature (Hayashi [1960]; O’Dor et al. [1982]; Sakurai et al. [1995]). It can affect the development and growth of the embryos, causing malformations or even the death.

**Figure 4:**
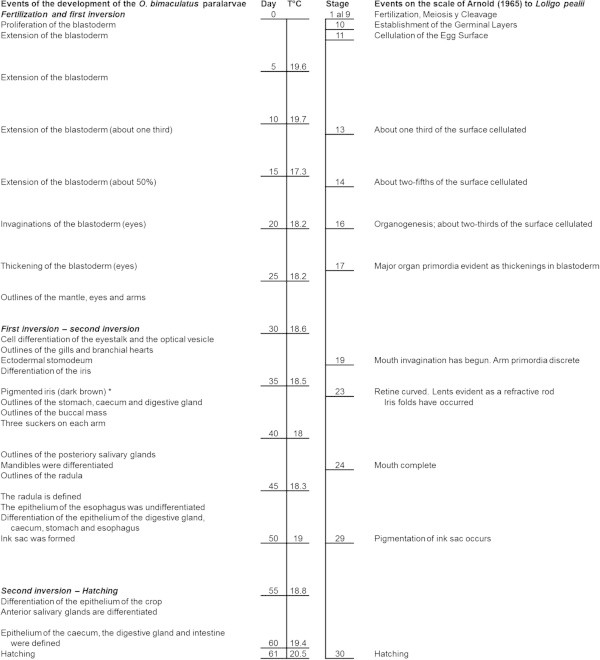
**Embryonic development of** ***Octopus bimaculatus*** **during 61 days incubation counted from the egg laying day.** The average temperature registered per five-day periods is indicated. The table compares the embryonic development with the scale proposed for the development of *L. pealii* (Arnold [1965]). *Observed *in vivo*.

The embryonic development of *O. bimaculatus* can be grouped in three stages: a) from fertilization to first inversion, in which the germinal layers are developed and the embryo prepares itself for organogenesis; b) from first inversion to second inversion, when the development of the organs of the anterior region of the digestive system occurs and the primordiums of most of the posterior region appears; and c) from second inversion to hatching, in which the organs of the posterior region of the digestive system develop, and it ends when the paralarvae hatch (Figure 4).

In the early development of *O. bimaculatus*, the mantle is the first differentiated structure. Later, the invaginations and then the thickening of the cellular layer that will become the optic vesicle and the eyes are observed. Simultaneously, the primordiums of the arms are formed on the equatorial constriction of the embryo. This sequence in the development is similar to that described for *Loligo duvauceli*, *Sepioteuthis arctipinnis*, *Octopus aegina* and *Euprymna scolopes* (Asokan and Kakati [1991]; Alagarswami [1966]; Ignatius and Srinivasan [2006]; Lee et al. [2009]). However, these studies only describe the external structures of the embryo and they do not make reference to the internal organs except those that are visible through the skin, like the gills and the branchial hearts. In these studies, the analysis of the embryonic development was performed solely by direct observation through the egg corion.

In general, the pattern of the embryonic development of *O. bimaculatus* is similar to that described by Arnold ([1965]) for *Loligo pealei* (Figure 4). The formation of the ectodermal stomodeum in the embryo of *O. bimaculatus* agrees with stage 19 in the scale of Arnold ([1965]) for *L. pealei*. This invagination appears in both studies once about 55% of the total incubation time passed. The complete formation of the buccal mass (44 DPL) agrees with stage 24, which in both studies corresponds to little more than 70% of the eggs incubation time. Nevertheless, the chronology of the ontogeny of some organs differs, like the eyes, which matured earlier in the *O. bimaculatus* paralarvae (38 DPL), when little more than 50% of the incubation time had passed, in comparison with *L. pealei* that matured in stage 25, which is equivalent to more than 80% of the incubation time. It is important to emphasize that the scale of Arnold ([1965]) was constructed based on the classification and description of the external anatomy changes of the embryo; therefore, it does not include the formation of the organs involved in the digestion, except the buccal mass.

In relation to hatched paralarvae, it was observed that the buccal mass is surrounded by a sanguineous sine that allows it to freely rotate during the bite action (Boyle et al. [1979]). The buccal mass is fortified with two mandibles, which, according to Lowenstam et al. ([1984]), are constituted of chitin hardened by means of mineral deposits like calcite and magnesite. In addition they are surrounded with muscular fibers for support and movement facilitation (Heinrich [1904]; Kear [1994]; Boucaud-Camou and Roper [1998]). The radula is exclusive of the mollusks, except bivalves and scaphopodes, and its structure is used for systematic classification (Aldrich et al. [1971]). In coleoids, the radula has 7 to 9 rows of teeth (Nixon [1968]; Aldred et al. [1983]; Mangold and Bidder [1989]), whereas in *O. bimaculatus* paralarvae, a row is composed of 30 longitudinal teeth positioned parallel to the longitudinal axis of the radula.

The location and the tissue structure of the anterior and posterior salivary glands are similar to those described in other young and adult cephalopods (Budelmann et al. [1997]; Boucaud-Camou and Roper [1998]). The diversity of cellular types observed in the posterior salivary glands of the *O. bimaculatus* paralarvae could be related to a diversity of secretions and indicates that newly hatched paralarvae have the capacity to produce different kinds of substances. Although the staining technique used reveals a diversity of cellular types, it was not possible to establish the nature of the secretions. Budelmann et al. ([1997]) mention that the mucus of the posterior salivary glands secretion in adult octopus contains proteoglycan acids and neutral, toxins, hyaluronidase and proteolytic enzymes, in addition to cardio excitatory and vasodilators like Ach, 5-HT transmitters and some catecholamine, that altogether serve to immobilize and paralyze the prey. Authors also mention that the anterior salivary glands contain neutral glycoproteins with SH and S-S groups, sialic acid, dipeptidase and hyaluronidase, that probably serve to liquefy viscous secretions of the rear salivary glands.

Young ([1965]) and Ducros ([1972]) indicate that in the adult octopods, the anterior salivary glands are profusely innervated by the sub-radula ganglion and the upper buccal lobe. It is important to mention that the current study was made with newly hatched paralarvae and that a specific technique for the nervous tissue identification was not used, making it difficult to identify in which stage of the paralarvae development the neural connection occurs.

Based in the histological constitution of the stomach of the *O. bimaculatus* paralarvae, it is likely that the stomach is the site where mechanical mixing of food is performed. Boucaud-Camou ([1977]) indicates the presence of an enzyme-rich fluid (pH 5–5.8) from the digestive gland containing proteases, lipases and amylases, which could assist the mixing and digestion of food. The globet cells that are characteristic of the stomach epithelium of adult cephalopods (Budelmann et al. [1997]) were not observed in the spotted octopus paralarvae, probably because the stomach is not yet completely developed. In this context, Boucaud-Camou and Roper ([1998]) indicate that the anterior region of the digestive system of *Rhynchoteuthion* paralarvae is more developed than the posterior region.

The caecum histological structure of *O. bimaculatus* paralarvae is similar to that described in adult Coleoids (Mangold and Bidder [1989]) and in *Rhynchoteuthion* paralarvae (Boucaud-Camou and Roper [1998]), since prominent folds in the mucous are observed. However these studies made no reference to the presence of cilia as we observed in the epithelium of the caecum of *O. bimaculatus* paralarvae, which can be an indicator of the degree of organ maturity. In the adults of *Illex argentinus*, the caecum has two sections, one of spiral structure and the other in sac form; both have a ciliate epithelium. These sections perform different biochemical functions: the spiral is a secreting type, while the region of the sac is digestive (Ivanovic and Brunetti [2002]).

In newly hatched paralarvae, the remaining amount of yolk was so small in contrast to a benthonic juvenile like *O. maya* (Moguel et al. [2010]) that it was not observable in the analyzed samples. The yolk is an energy source that allows the paralarvae to survive during the first days of free life, before they start exogenous food ingestion (Moguel et al. [2010]). The structure of the epithelium of the tubules that form the digestive gland of *O. bimaculatus* paralarvae is similar to other adult octopus (Budelmann et al. [1997]) and to the paralarvae of *Rhynchoteuthion* (Boucaud-Camou and Roper [1998]). Mangold and Bidder ([1989]) mentioned that the tubules have two types of cells, basal and digestive. The digestive cells undergo structural changes which have been related to digestion suggesting that the gland itself is a dynamic structure that experiences proliferation cycles, growth and cell differentiation (Ivanovic and Brunetti [2002]). The most notable feature of these cells is the presence of numerous vacuoles with appearance and different contents depending on the function they perform (Boucher-Rodoni [1976]; Mangold and Bidder [1989]). Basal cells containing vacuoles have storage sites of Fe, Cu, Ca and heavy metals (Budelmann et al. [1997]). While appendixes of the digestive gland are the site of absorption of some nutrients such as amino acids and carbohydrates (Boucher-Rodoni and Mangold [1977]), also they are involved in osmoregulation processes and enzyme secretion (Wells and Wells [1989]).

Budelmann et al. ([1997]) reported that the membrane which surrounds the digestive gland in cephalopods adults is composed of collagen and muscular fibers; however, the color of the tissues that resulted from the stain probably indicates that this structure in *O. bimaculatus* paralarvae is still immature at this stage.

The intestine of paralarvae of *O. bimaculatus* has a folded columnar epithelium lacking of globet cells, which are characteristic of the intestine epithelium in the adult squid *I. argentinus* (Ivanovic and Brunetti [2002]). This organ serves as a channel for the passage of waste, but also is the site where absorption of small molecules, particularly water, occurs (Ivanovic and Brunetti [2002]).

In newly hatched marine fish larvae, the digestive tract is a straight undifferentiated tube placed dorso-ventral position with respect to the viteline sac. The anatomical differentiation happens at the time that the larvae has consumed its viteline reserve and initiates the exogenous food ingestion (Gonovi et al. [1986]; Kuzmina and Gelman [1998]). In contrast, in the *O. bimaculatus* paralarvae, the organs involved in the ingestion and digestion of food are already differentiated in a similar arrangement to an adult organism. Because of this similarity, it is important to study with greater detail the different structures of the digestive system to identify and discern their degree of maturity, which could explain their capacity to digest the food and absorb the nutrients.

The present research constitutes the first description at histological level of the ontogeny of the digestive system of the *O. bimaculatus* paralarvae, including details of the different phases of the embryo development, as well as the integration and development of the diverse organs involved in the digestive processes. This information provides the bases to understand the organization and functionality of the digestive system of paralarvae during the first days after hatching, and that could be used in the future studies of the digestive physiology of this species.
